# A complementary approach for genetic diagnosis of inborn errors of immunity using proteogenomic analysis

**DOI:** 10.1093/pnasnexus/pgad104

**Published:** 2023-03-28

**Authors:** Fumiaki Sakura, Kosuke Noma, Takaki Asano, Kay Tanita, Etsushi Toyofuku, Kentaro Kato, Miyuki Tsumura, Hiroshi Nihira, Kazushi Izawa, Kanako Mitsui-Sekinaka, Ryo Konno, Yusuke Kawashima, Yoko Mizoguchi, Shuhei Karakawa, Seiichi Hayakawa, Hiroshi Kawaguchi, Kohsuke Imai, Shigeaki Nonoyama, Takahiro Yasumi, Hidenori Ohnishi, Hirokazu Kanegane, Osamu Ohara, Satoshi Okada

**Affiliations:** Department of Pediatrics, Hiroshima University Graduate School of Biomedical and Health Sciences, 1-2-3 Kasumi, Minami Ward, Hiroshima 734-8551, Japan; Department of Pediatrics, Hiroshima University Graduate School of Biomedical and Health Sciences, 1-2-3 Kasumi, Minami Ward, Hiroshima 734-8551, Japan; Department of Pediatrics, Hiroshima University Graduate School of Biomedical and Health Sciences, 1-2-3 Kasumi, Minami Ward, Hiroshima 734-8551, Japan; Department of Pediatrics and Developmental Biology, Graduate School of Medical and Dental Sciences, Tokyo Medical and Dental University (TMDU), 1-5-45 Yushima, Bunkyo City, Tokyo 113-0034, Japan; Department of Pediatrics and Developmental Biology, Graduate School of Medical and Dental Sciences, Tokyo Medical and Dental University (TMDU), 1-5-45 Yushima, Bunkyo City, Tokyo 113-0034, Japan; Department of Pediatrics, Kyoto University Graduate School of Medicine, 54 Shogoin Kawaharacho, Sakyo Ward, Kyoto City 606-8507, Japan; Department of Pediatrics, Hiroshima University Graduate School of Biomedical and Health Sciences, 1-2-3 Kasumi, Minami Ward, Hiroshima 734-8551, Japan; Department of Pediatrics, Kyoto University Graduate School of Medicine, 54 Shogoin Kawaharacho, Sakyo Ward, Kyoto City 606-8507, Japan; Department of Pediatrics, Kyoto University Graduate School of Medicine, 54 Shogoin Kawaharacho, Sakyo Ward, Kyoto City 606-8507, Japan; Department of Pediatrics, National Defense Medical College, 3-2 Namiki, Tokorozawa City, Saitama 359-8513, Japan; Kazusa DNA Research Institute, 2-6-7 Kazusakamatari, Kisarazu City, Chiba 292-0818, Japan; Kazusa DNA Research Institute, 2-6-7 Kazusakamatari, Kisarazu City, Chiba 292-0818, Japan; Department of Pediatrics, Hiroshima University Graduate School of Biomedical and Health Sciences, 1-2-3 Kasumi, Minami Ward, Hiroshima 734-8551, Japan; Department of Pediatrics, Hiroshima University Graduate School of Biomedical and Health Sciences, 1-2-3 Kasumi, Minami Ward, Hiroshima 734-8551, Japan; Department of Pediatrics, Hiroshima University Graduate School of Biomedical and Health Sciences, 1-2-3 Kasumi, Minami Ward, Hiroshima 734-8551, Japan; Department of Pediatrics, Hiroshima University Graduate School of Biomedical and Health Sciences, 1-2-3 Kasumi, Minami Ward, Hiroshima 734-8551, Japan; Department of Pediatrics, National Defense Medical College, 3-2 Namiki, Tokorozawa City, Saitama 359-8513, Japan; Department of Pediatrics, National Defense Medical College, 3-2 Namiki, Tokorozawa City, Saitama 359-8513, Japan; Department of Pediatrics, Kyoto University Graduate School of Medicine, 54 Shogoin Kawaharacho, Sakyo Ward, Kyoto City 606-8507, Japan; Department of Pediatrics, Gifu University Graduate School of Medicine, 1-1 Yanagido, Gifu City 501-1112, Japan; Department of Pediatrics and Developmental Biology, Graduate School of Medical and Dental Sciences, Tokyo Medical and Dental University (TMDU), 1-5-45 Yushima, Bunkyo City, Tokyo 113-0034, Japan; Kazusa DNA Research Institute, 2-6-7 Kazusakamatari, Kisarazu City, Chiba 292-0818, Japan; Department of Pediatrics, Hiroshima University Graduate School of Biomedical and Health Sciences, 1-2-3 Kasumi, Minami Ward, Hiroshima 734-8551, Japan

**Keywords:** proteomics, inborn errors of immunity, targeted RNA sequencing, genetic diagnosis

## Abstract

Advances in next-generation sequencing technology have identified many genes responsible for inborn errors of immunity (IEI). However, there is still room for improvement in the efficiency of genetic diagnosis. Recently, RNA sequencing and proteomics using peripheral blood mononuclear cells (PBMCs) have gained attention, but only some studies have integrated these analyses in IEI. Moreover, previous proteomic studies for PBMCs have achieved limited coverage (approximately 3000 proteins). More comprehensive data are needed to gain valuable insights into the molecular mechanisms underlying IEI. Here, we propose a state-of-the-art method for diagnosing IEI using PBMCs proteomics integrated with targeted RNA sequencing (T-RNA-seq), providing unique insights into the pathogenesis of IEI. This study analyzed 70 IEI patients whose genetic etiology had not been identified by genetic analysis. In-depth proteomics identified 6498 proteins, which covered 63% of 527 genes identified in T-RNA-seq, allowing us to examine the molecular cause of IEI and immune cell defects. This integrated analysis identified the disease-causing genes in four cases undiagnosed in previous genetic studies. Three of them could be diagnosed by T-RNA-seq, while the other could only be diagnosed by proteomics. Moreover, this integrated analysis showed high protein–mRNA correlations in B- and T-cell-specific genes, and their expression profiles identified patients with immune cell dysfunction. These results indicate that integrated analysis improves the efficiency of genetic diagnosis and provides a deep understanding of the immune cell dysfunction underlying the etiology of IEI. Our novel approach demonstrates the complementary role of proteogenomic analysis in the genetic diagnosis and characterization of IEI.

**Significance Statement**

Genetic diagnosis plays a central role in the clinical management of patients with inborn errors of immunity (IEI). However, the diagnostic yield for IEI based on the sequencing of germline DNA is still low and is estimated to be approximately 30%. This study shows the utility of integrated analysis with proteomics and targeted RNA sequencing (T-RNA-seq) of peripheral blood mononuclear cells. We identified the molecular cause and immune cell defects in patients with IEI, increasing the diagnostic yield by 6%. Notably, even in cases missed by T-RNA-seq, proteomics could identify the genetic etiology of the disease, suggesting the pivotal role of proteomic analysis in diagnosing IEI. Our novel approach improves the efficiency of the genetic diagnosis and elucidates the pathogenesis of IEI.

Patients with inborn errors of immunity (IEI), previously known as primary immunodeficiency disorders, demonstrate increased susceptibility to infectious diseases, autoimmunity, autoinflammatory diseases, allergies, and malignancies (1). These conditions are generally caused by monogenic germline defects resulting in the dysfunction of encoded proteins. The latest classification of IEI from the International Union of Immunological Societies (IUIS) Expert Committee includes 485 genes as genetic etiologies of IEI, representing an increase of 55 genes since the 2019 IUIS update (2). This breakthrough occurred predominantly due to the application of next-generation sequencing (NGS) technologies, such as targeted gene panel NGS (T-NGS), whole-exome sequencing (WES), or whole-genome sequencing (3–5). Genetic diagnosis plays a pivotal role in the clinical management in IEI patients because elucidating the molecular etiology paves the way for fundamental therapies; 34% of genetically diagnosed cases have distinct therapeutic options (5). However, the diagnostic yield of NGS for IEI is still low and is estimated to be approximately 30 to 40% (6, 5, 7–9). WES and T-NGS have several inherent limitations, explaining these undiagnosed cases. The most challenging of those limitations is the difficulty of interpreting variants of unknown significance (10, 11). Other drawbacks are the inability to detect variants in noncoding regions (12).

RNA sequencing (RNA-seq) has been well employed as one of the most valuable tools to study Mendelian disorders (10, 13), because it provides complementary information about the downstream consequences of genomic variants, such as variations in RNA abundance, allele-specific expression (ASE) and alternative splicing isoforms (13, 14). Especially the use of targeted RNA-seq (T-RNA-seq) is a well-established approach for investigating low-abundance transcripts or low-input RNA samples (15, 16) and is advantageous in studying IEI, in which the expression of disease-causing genes is often suppressed. Indeed, many studies on IEI have confirmed the effectiveness of RNA-seq or T-RNA-seq (17–21). However, the diagnostic yield of IEI remains in the 7.5–36% range for patients for whom T-NGS or WES is uninformative (10, 22, 23). One of the most significant current discussions regarding RNA-seq is the discordance of RNA and protein expression levels. The controversy about the relationship between protein abundance and its coding mRNA abundance has continued unabated due to the development of high-throughput technologies that simultaneously interrogate the global abundance of protein and mRNA (24–26).

More recently, researchers have shown an increasing interest in proteomics due to technological advances in mass spectrometry (MS)-based protein identification (27, 28). To date, more than 90% of the proteins corresponding to known protein-coding genes have been detected by MS-based proteomics (29). A recent literature review concluded that MS-based proteomics contributed substantially to our understanding of innate immunity (30). This review also pointed out that overcoming problems associated with low abundance of cellular fractions and high abundance of degradative proteases will be required to obtain an unbiased and comprehensive protein profile. Since hematopoietic cells form the basis of the pathogenesis of IEI, expression analysis of peripheral blood mononuclear cells (PBMCs) is useful to determine the molecular pathogenesis. However, previous PBMC proteomics studies using data-independent acquisition (DIA)-MS, which provides higher sensitivity, higher protein coverage, and greater reproducibility than classic data-dependent acquisition, have identified only approximately 3000 proteins (31–33). Considering that patients with IEI have a variety of immune cell defects and disease-causing protein defects, more comprehensive proteomic data are needed to gain rational insights into the molecular mechanisms underlying aberrant immune systems. A few studies have applied proteomics to the genetic diagnosis of IEI (34, 35). However, the current study is the first to examine the utility of in-depth proteomics in integrated analysis in combination with T-RNA-seq.

Here, we propose a state-of-the-art method for diagnosing IEI, providing notable insights into the pathogenesis of IEI. Our single-shot DIA-MS approach, which was high-throughput and cost-effective, enabled proteomic analysis of PBMCs at greater depth. Furthermore, this improved analytical depth achieved protein coverage nearly equivalent to the depth of transcriptome analysis by RNA-seq and allowed integrated analysis with T-RNA-seq. This study aims to highlight the complementary role of integrated analysis of proteomics and T-RNA-seq to canonical genomic analysis in determining the molecular pathogenesis of IEI.

## Results

### In-depth proteomic data from PBMCs covered many IEI-related genes

**Fig. 1.**
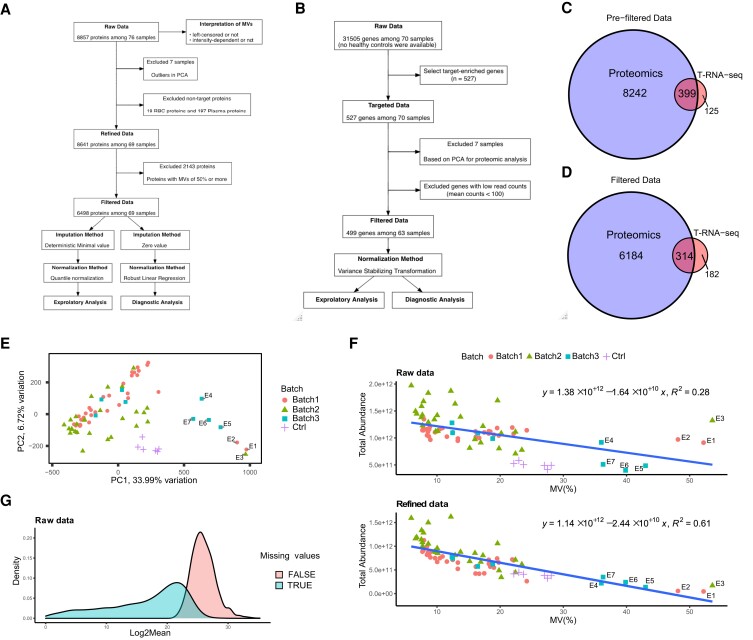
Overview of the initial processing of proteomics and RNA-seq data. A) Schematic diagram of proteomic analysis. DIA-MS yielded 8857 proteins from 70 IEI patients and six healthy donors. Interpretation for MVs was performed with raw data. MVs filtering was performed on 8641 protein data points among 69 patients, resulting in a filtered dataset of 6498 proteins. Downstream analysis was performed using two methods, each with optimal MVs imputation and normalization. B) Schematic diagram of targeted RNA-seq (T-RNA-seq). No RNA-seq data from healthy controls were available. Quality control was performed with data from 527 target-enriched genes, yielding filtered data for 499 genes among 63 cases. C) Venn diagrams of genes identified by proteomics and T-RNA-seq (8641 vs. 524). The blue circle reflects the proteomics data excluding RBC and plasma proteins, and the red circle reflects the targeted genes. Among the 527 targeted genes in T-RNA-seq, four noncoding RNAs were excluded. D) Venn diagram for filtered data (6498 vs. 496). Three noncoding RNAs were excluded from 499 genes. E) PCA of raw proteomic data showing the eligibility of the data. Batches are indicated by shape and color. The *x*-axis shows the first principal component (PC1), and the *y*-axis shows the second principal component (PC2). Only excluded samples are labeled (E1 to E7). F) Correlation of total protein abundance and MVs proportion. Protein intensity excluding RBCs and plasma proteins (targeted protein) is shown in the top figure, and the raw protein abundance is shown in the bottom figure. The linear regression, its formula, and R-squared values are shown in the figure. The shape and color coding are the same as in E). G) Density plot represents the distribution of protein abundance with or without MVs. The blue area contains proteins with MVs, and the red area contains proteins without MVs.

The current study encompassed a cohort of 70 patients diagnosed with IEI but without a known genetic etiology. Of these, 48 patients underwent WES, and the remaining 22 underwent T-NGS of a 400 IEI gene panel. Prior genetic analysis was conducted based on criteria established by the American College of Medical Genetics and Genomics (ACMG), along with the patients’ phenotypes and the disease’s inheritance mode. However, no pathogenic variants were identified that satisfied these criteria. (*SI Appendix*, Table S1). The first set of analyses examined the eligibility of the proteomic data. The initial processing of the proteomic data identified 8857 proteins; after data optimization, 6498 (73% of detected proteins) proteins from 63 IEI patients and six healthy controls (HCs) (91% of all participants) were retained for downstream analysis (Fig. 1A, *SI Appendix*, Dataset S1). T-RNA-seq provided data for 527 IEI-related genes, almost all of which were highly enriched, in 63 cases (Fig. 1A, *SI Appendix*, Fig. S1A and Dataset S2). We then removed the genes with total read counts of less than 1000, leaving T-RNA-seq data for 499 genes in 63 cases (Fig. 1B). Surprisingly, the refined proteomic data, which excluded nontarget proteins such as plasma and RBCs, identified 8641 proteins from PBMCs, covering 80% of the genes in T-RNA-seq (399 out of 496 genes; three noncoding genes were removed) (Fig. 1C). Although filtering the data to remove proteins with high missing values (MVs) reduced that coverage to 63% (314 out of 496 genes) (Fig. 1D), our proteomic data still maintained high coverage. Overall, these results show that our proteomic analysis covered many known IEI genes and allowed us to perform integrated mRNA–protein analysis.

### Detailed interpretation of proteomic data enabled optimization of initial processing

We next performed data interpretation to ensure the validity and reproducibility of the proteomic data. We excluded seven samples with a higher proportion of MVs from this study (E1 to E7) based on the PCA for raw data (Fig. 1E). Regarding the assessment of MVs with linear regression, the refined protein abundance and the proportion of MVs showed a negative correlation, with an R-squared value of 0.61 (Fig. 1F), which was markedly higher than that of the total protein abundance including nontarget proteins. Moreover, the distribution of mean expression levels was biased toward lower levels for proteins with MVs compared to those without MVs (Fig. 1G). These results indicated that MVs were abundance-dependent and left-censored. Another significant aspect of this result is that the difference in R-squared values between total proteins and targeted proteins indicates that the dominance of nontarget proteins overwhelmed the abundance of the proteins of interest and increased the number of MVs (Fig. 1F). Regarding MVs being left-censored data, we adopted small-value imputation methods separately for exploratory and diagnostic analyses. Considering that the MVs were below the detection limit, the zero-value method was adapted for the diagnostic analysis. Meanwhile, the minimum deterministic method was selected for the exploratory analysis because distance-based clustering, such as the *k*-means method, is not sensitive to zero value, especially in cases with a small *k* value. Then, based on the results of the NormalyzerDE comparison, we normalized the imputed data with quantile normalization and robust linear regression normalization (*SI Appendix*, Fig. S1B). Similarly, we normalized the T-RNA-seq data with the variance stabilizing transformation method (*SI Appendix*, Fig. S1C). In summary, our data interpretation approach revealed the nature of the MVs and allowed data optimization (*SI Appendix*, Dataset S1).

### Diagnostic analysis identifies disease-causing protein

**Fig. 2.**
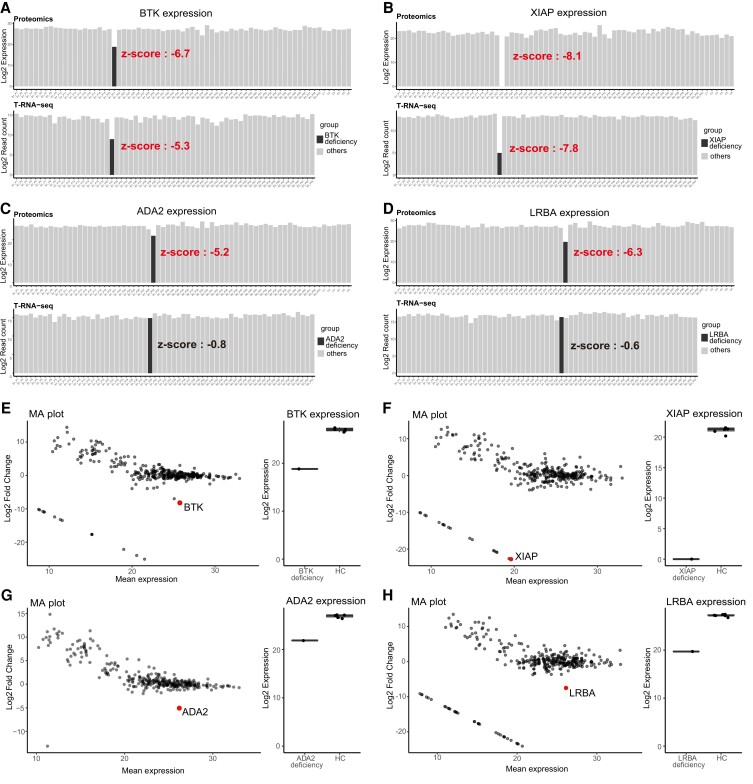
Diagnostic analysis of disease-causing genes and Correlation analysis of proteomics and T-RNA-seq. A–D) The bar plot shows the distribution of disease-causing protein and mRNA expression levels per sample. Throughout the figure, protein expression is shown at the top and mRNA expression is shown at the bottom; the case is shown in black, and other samples are shown in gray. A) BTK deficiency, B) XIAP deficiency, C) ADA2 deficiency, and D) LRBA deficiency. E–H) Decreased expression of disease-causing proteins compared to healthy controls (HCs). MA plot shows that the disease-causing protein is prominently downregulated (left panel). The *x*-axis shows the log mean expression of each protein, and the *y*-axis shows the log fold change of protein expression between the patient and HCs. The plots shown on a straight line in the lower left of the figure are proteins showing the MVs in the patients. The right panel shows the distribution of disease-causing protein expression in the patient and HCs. E) BTK deficiency, F) XIAP deficiency, G) ADA2 deficiency, and H) LRBA deficiency.

Our study allows direct comparison of protein and mRNA expression profiles because the data were generated from the same specimens. Therefore, we examined the utility of proteomic analysis in genetic diagnosis by comparing the protein and mRNA expression levels of 314 overlapping genes (*SI Appendix*, Dataset S3). We identified four cases where a proteomic analysis unveiled the disease-causing protein (Table S1). Bruton tyrosine kinase (BTK) deficiency (B1_P21) and X-linked inhibitor of apoptosis (XIAP) deficiency (B1_P22) exhibited impressive reductions in protein (*z*-scores; −6.7 and −8.1, respectively) and mRNA (*z*-scores; −5.3 and −7.8, respectively) (Fig. 2A and B), despite a lack of significant findings in the initial genomic analysis. In contrast, adenosine deaminase 2 (ADA2) deficiency (B1_P29) and LPS-responsive beige-like anchor protein (LRBA) deficiency (B2_P35) presented no reduction in mRNA expression (*z*-scores; −0.8 and −0.6, respectively) but a considerable reduction in protein expression (*z*-scores; −5.2 and −6.3, respectively) (Fig. 2C and D). In these cases, only monoallelic variants were identified in genome analysis, and no genetic diagnosis was made. Proteomic analysis thus provided unique information directly related to a definitive diagnosis in these two cases. In addition, the protein expression profiles of these four cases were compared to HCs as a means of making a diagnosis in a single case. Each disease-causing protein was highly expressed in HCs, while its expression was markedly decreased with log2-fold change <−5 in each patient, indicating a decrease of more than 1/32 from the average expression (Fig. 2E–H, Dataset S3).

### Validation analysis links the results of the diagnostic analysis to the clinical diagnosis

Since genetic diagnosis is based on genomic variants, we performed further analysis to validate the results of our diagnostic analysis. The results are summarized in Table 1. In a BTK-deficient case, the intronic variant of c.−196+1G>T was detected by follow-up genomic analysis. This 5′-UTR was not only a splice site but also contained a number of transcriptional regulators that may have explained the results of the diagnostic analysis (*SI Appendix*, Fig. S2), but detailed pathogenicity is currently under analysis. In an XIAP-deficient case, Western blotting and RT–PCR also showed decreased protein and mRNA expression levels. In addition, targeted sequencing covering the entire *XIAP* region identified a large deletion containing a noncoding exon with promoter activity. These results were previously reported by Sbihi et al., and “patient 2” corresponded to this case (36). In an ADA2-deficient case, decreased ADA2 activity was observed in the patient and was a supportive laboratory finding. Some results have already been reported by Nihira et al., and “patient 2” corresponded to this case (37). T-RNA-seq revealed aberrant splicing in this case (*SI Appendix*, Fig. S3A). The results of LeafCutter show that the aberrant junction is specific to this case (*SI Appendix*, Fig. S3A). Moreover, variant calling on T-RNA-seq revealed the intronic variant of c.972+102T>G, which generated an abnormal splicing profile, and the known missense variant led to ASE, with unequal expression between the wild-type and mutant alleles (20% and 80%, respectively) (*SI Appendix*, Fig. S3B). Given that aligned reads harbored missense and intronic variants separately, compound heterozygous variants in *ADA2* were the cause of the disease. In LRBA deficiency cases, the results of the diagnostic analysis are under verification. However, the patient showed various autoimmune abnormalities consistent with the phenotype of LRBA deficiency. In addition, we observed supportive laboratory findings of decreased CTLA4 expression in Tregs and decreased LRBA expression, as determined by Western blotting. These results suggest that our diagnostic analysis can contribute to clinical diagnosis. In summary, although genetic diagnosis was possible in three patients by T-RNA-seq alone, integrated analysis with proteomics enabled genetic diagnosis in one additional patient, increasing the efficiency of genetic diagnosis by 6% in patients who could not be diagnosed by genetic analysis (Table 2).

**Table 1.** Summary of the results of the diagnostic analysis and its validation analysis.

Patient ID	Pathogenic gene	Variants detected in prior genetic analysis	Results of diagnostic analysis	Genomic variants detected in follow-up analysis	Supportive laboratory findings
B1_P21	*BTK*	No pathogenic variants	Decreased protein and mRNA expression levels	c.-196+1G>T (variant in splice-site and cis-regulatory region)	B-cell defects via flow cytometry (0.1% of total lymphocytes)
B1_P22	*XIAP*	No pathogenic variants	Decreased protein and mRNA expression levels	Large deletion in promoter region (36)	Decreased XIAP expression in RT–PCR and WB (36)
B1_P29	*ADA2*	c.982G>A: p.Glu328Lys (heterozygous)	Decreased expression only at the protein level	Aberrant splicing with intoronic variant of c.972+102T>G Allele specific expression	Decreased ADA2 activity (37)
B2_P35	*LRBA*	c.1219_1220del: p.Leu408Valfs*7 (heterozygous)	Decreased expression only at the protein level	Being analyzed	Decreased CTLA4 expression in Tregs Decreased LRBA expression via WB

RT–PCR, reverse transcription PCR; WB, Western blotting; CTLA4, cytotoxic T-lymphocyte associated protein 4; Tregs, regulatory T cells.

**Table 2.** Diagnostic efficiency in undiagnosed patients using WES or T-NGS.

Method	Number of diagnosed patients	The increase in diagnostic efficiency	Notes
T-RNA-seq	3(BTK deficiency, XIAP deficiency, ADA2 deficiency)	4%	ADA2 deficiency could possibly be diagnosed via T-RNA-seq alone by identifying aberrant splicing
Proteomics	1(LRBA deficiency)	2%	• Proteomics was the only diagnostic evidence of LRBA deficiency
			• Proteomics provided supportive findings at the protein level in BTK, XIAP, and ADA2 deficiency
T-RNA-seq + Proteomics	4	6%	

### The protein and mRNA expression levels of B- and T-cell-specific genes show strong correlations

**Fig. 3.**
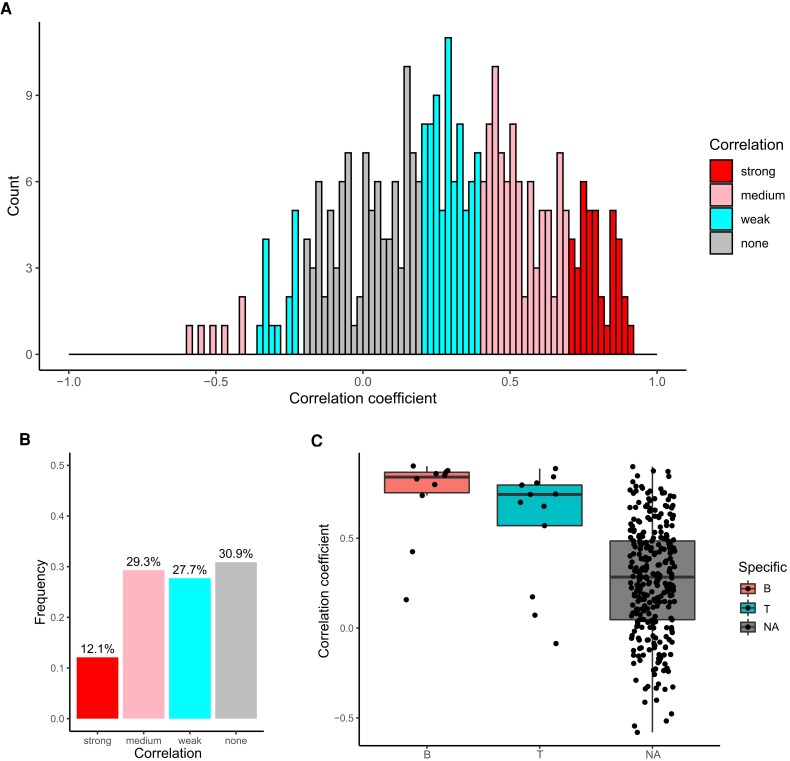
Correlation analysis of IEI-related genes. A) Spearman correlation coefficients of protein and mRNA levels for genes identified by proteomics and T-RNA-seq. The color scale reflects the degree of correlation, with red bars indicating a strong correlation, pink bars indicating a moderate correlation, blue bars indicating a weak correlation, and gray bars indicating no correlation. B) The bar chart indicates the frequency of each degree of correlation among the 314 genes. The color coding is the same as in Figure E. C) Jitter boxplot showing the distribution of the correlation coefficients for cell-specific genes. The color scale reflects specific cell types, with red indicating B cells, blue indicating T cells, and gray indicating genes that do not correspond to a specific cell type (NA).

Considering that a discrepancy between protein and mRNA expression of the disease-causing gene was noted in two cases in our diagnostic analysis, we systematically analyzed the correlation between protein and mRNA levels. We first calculated Spearman’s correlation coefficients for 314 genes identified by both proteomics and T-RNA-seq among our 63 patients (Fig. 3A and *SI Appendix*, Dataset S4) and found that the median correlation was 0.29 (interquartile range of 0.07–0.52). Furthermore, the distribution of correlation coefficients indicates that more than half of the genes have an absolute correlation coefficient of less than 0.4, that is, weak or no correlation (Fig. 3B). These results indicate a discrepancy between protein and mRNA expression levels. Because the genes targeted in T-RNA-seq included the immune-cell-specific genes used as cell markers, we also compared protein–mRNA correlations of B-, T-, and NK-cell-specific genes. We identified 10 B-cell- and 13 T-cell-specific genes among the 314 genes but no NK-cell-specific genes. Interestingly, the correlation coefficients for B-cell-specific and T-cell-specific genes were 0.84 and 0.74, respectively, showing a strong correlation (Fig. 3C).

### Exploratory analysis of B-cell-specific proteins enables the identification of B-cell-deficient cases

**Fig. 4.**
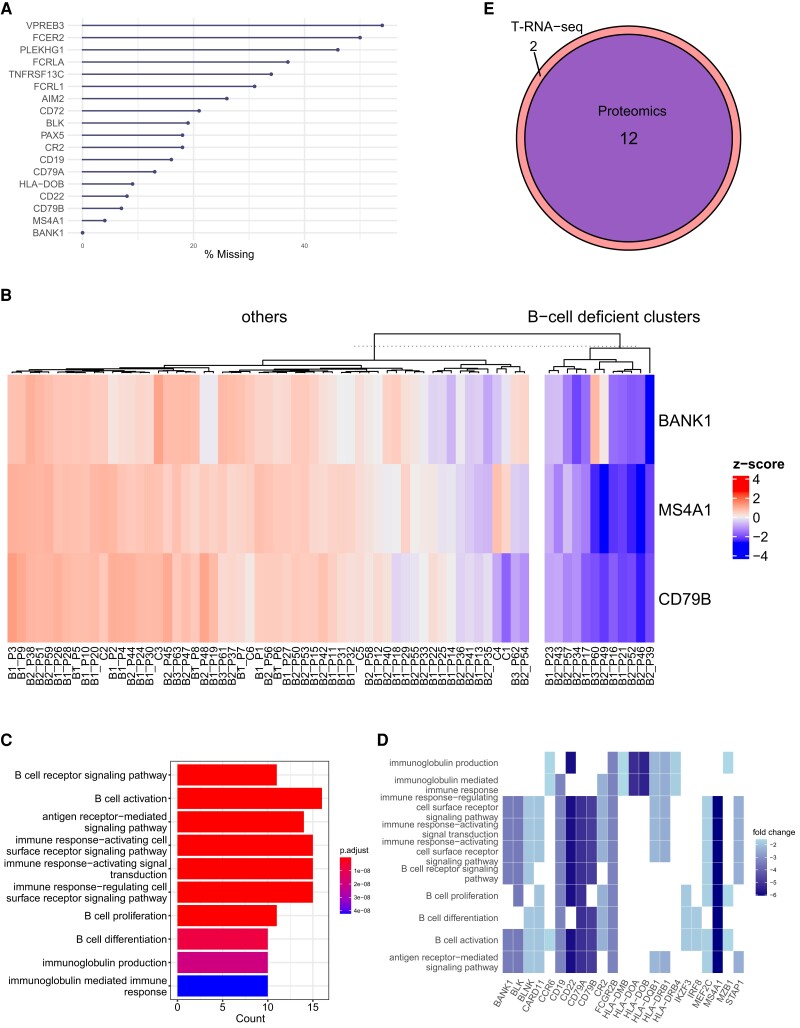
Exploratory analysis of B-cell dysfunction. A) Missing value analysis of B-cell-specific proteins. The *x*-axis shows the percentage of missing values among 69 samples. B) The heatmap of *k*-means clustering shows cluster segregation with decreased expression of B-cell-specific proteins. The color scale reflects the *z* score, with red indicating a positive value and blue a negative value. C) Top 10 enriched GO terms for proteins downregulated in the comparison of the B-cell-deficient cluster and others. The color scale reflects the adjusted *P* value of each GO term. D) Heatmap of proteins associated with the top 10 enriched GO terms. The color scale shows the log fold change; the darker the color tone is, the lower the expression. E) Venn diagram showing that all B-cell-deficient clusters identified by proteomics are included in the clusters identified by RNA-seq. The blue area indicates proteomics, and the red area indicates RNA-seq.

Based on the strong correlation of proteomic and T-RNA-seq data in B and T cells detected in the current study, we investigated whether proteomic analysis could discriminate the population with immune cell defects, which play a pivotal role in the pathogenesis of IEI. We thus analyzed proteomic data with *k*-means clustering based on immune cell-specific protein profiles (*SI Appendix*, Dataset S5). First, we extracted 18 B-cell-specific proteins (based on public databases) from our proteomic data (Fig. 4A) and selected three according to the criteria described in the Methods (see “Exploratory analysis of B- and T-cell deficiency”). We then segregated 12 cases into B-cell-deficient cluster by *k*-means clustering (Fig. 4B). Interestingly, eight out of 12 cases categorized as B-cell-deficient cluster were classified in IUIS category 3 as “predominantly antibody deficiencies,” and five of them showed apparent B-cell defects in flow cytometry (FCM) analysis (*SI Appendix*, Table S2). To validate the clustering results, we performed GO analysis of significantly downregulated genes (log-fold-change <−1.5 and *p*-value <0.05) in a two-group comparison (B-cell-deficient clusters vs. others). The results showed that many genes involved in B-cell function were strongly downregulated in the B-cell-deficient group, even in the total protein profile, suggesting that the clustering results were valid (Fig. 4C and D). For further validation of the proteomics results, we compared the results with those of T-RNA-seq (*SI Appendix*, Fig. S4A). The 14 B-cell-deficient cases identified by T-RNA-seq included all 12 B-cell-deficient cases in the proteomics, indicating the strong protein–mRNA correlation of B-cell-specific genes (Fig. 4E, *SI Appendix*, Table S3). In summary, PBMC proteomics enabled the identification of cases with B-cell dysfunction based on their quantitative changes.

### Comprehensive protein analysis reveals T-cell dysfunction in diverse disease types, and T-RNA-seq reveals diversity in the expression profiles of T-cell-specific genes

**Fig. 5.**
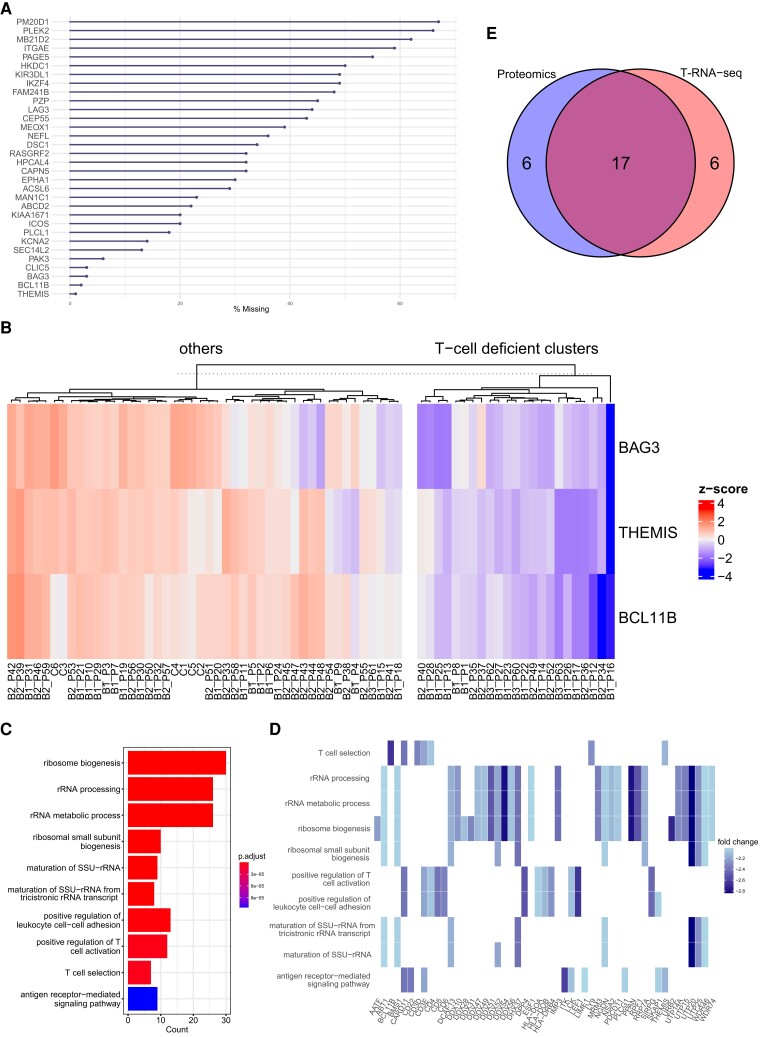
Exploratory analysis of T-cell dysfunction. A) Missing value analysis of T-cell-specific proteins. The *x*-axis indicates the percentage of missing values among the 69 samples. B) The heatmap of *k*-means clustering shows cluster segregation with decreased expression of T-cell-specific proteins. The color scale reflects the *z* score, with red indicating a positive value and blue a negative value. C) Top 10 enriched GO terms for proteins downregulated in the comparison of the T-cell-deficient cluster and others. The color scale reflects the adjusted *P* value of each GO term. D) Heatmap of proteins associated with the top 10 enriched GO terms. The color scale reflects the log fold change; the darker the color tone is, the lower the expression. E) Venn diagram of T-cell-deficient clusters identified by proteomics and RNA-seq. The blue area indicates proteomics, and the red area indicates RNA-seq.

Next, we examined T-cell dysfunction, which provides a helpful benchmark for the validity of our study because T-cell function is diverse, and its dysfunction is implicated in the pathogenesis of various forms of IEI. Our proteomic analysis identified 32 T-cell-specific proteins (Fig. 5A and *SI Appendix*, Dataset S5), and clustering analysis identified 23 cases of T-cell deficiency (Fig. 5B). The Results show that half of the T-cell-deficient cluster are either combined immunodeficiency or IUIS category 4 as “diseases of immune dysregulation,” in which T-cell dysfunction is the predominant pathological feature (*SI Appendix*, Table S4). Most of the remaining cases were suggested to be common variable immune deficiency (CVID), but only three of them were also classified as B-cell deficient. On the other hand, a case of X-linked agammaglobulinemia, which presents as a pure B-cell defect, was not included in the T-cell-deficient cluster, indicating the heterogeneous nature of CVID. GO analysis of the proteins downregulated in the T-cell-deficient cluster vs. others showed that terms involved in ribosome biogenesis and ribosomal RNA were highly enriched (Fig. 5C), and the protein expression of those involved in T-cell function was also suppressed to the same extent (Fig. 5D). In contrast to the analysis of B-cell deficiency, only 17 T-cell-deficient cases in T-RNA-seq matched the cluster in the proteomic analysis (Fig. 5E, and *SI Appendix*, Table S5). This is an unexpected result but is attributed to the fact that clustering based on T-cell-specific genes was highly variable (*SI Appendix*, Fig. S4B), and the elbow point, which indicates the optimal number of clusters, was uniquely greater than a value of two in T-cell analysis of T-RNA-seq (*SI Appendix*, Fig. S5A and B). These results suggest that T-cell function in IEI is more complex than B-cell function, and in particular, the mRNA expression of T-cell-specific genes exhibits a diverse profile.

## Discussion

This study analyzed 63 patients with IEI through in-depth proteomic analysis of PBMCs, identifying 6498 proteins that covered 63% of the genes covered by the T-RNA-seq. The improved comprehensiveness and mRNA coverage allowed an integrated analysis of protein and mRNA and revealed the discrepancies between protein and mRNA expression levels. These findings demonstrate the importance of proteomic analysis and its role as a complement to RNA-seq for IEI. The most important clinically relevant result was that these gene expression analyses enabled genetic diagnosis in four cases, two of which could be diagnosed only by proteomic analysis. In addition, an integrated study with T-RNA-seq elucidated the genomic basis of the disease in one case. Another significant finding was that proteomic data allowed us to classify the cases of immune cell defects based on protein profiles specific to those cells. Exploratory analysis then revealed immune cell dysfunction in terms of comprehensive molecular interactions. These findings suggest that an integrated analysis of proteomics and T-RNA-seq facilitates the understanding of the pathogenesis and underlying immune cell defects in IEI cases.

One fascinating finding was that diagnostic analysis revealed the disease-underlying protein in four cases. Among them, BTK- and XIAP-deficient cases demonstrated a noticeable reduction in both protein and mRNA expression. Further analysis proved that these results were due to genomic variants in the promoter region. In contrast, ADA2- and LRBA-deficient cases exhibited discordance between protein and mRNA expression, where decreased expression was observed only at the protein level. In these cases, the identification of the lack of ADA2 activity and reduced LRBA expression in western blotting aided in the clinical diagnosis. Proteomic analysis thus provides essential information that contributes to clinical diagnosis. Moreover, T-RNA-seq for ADA2 deficiency showed ASE in genomic locations bearing missense variants which may trigger nonsense-mediated decay (NMD). This finding is consistent with previous findings by Rivas et al., who demonstrated that variants generating premature stop codons and predicted to trigger NMD were prone to demonstrate ASE (38). Nevertheless, NMD occurring in the allele of the intronic variant in ADA2 did not significantly affect the mRNA expression levels, and its pathological significance was identified via the decrease in protein expression levels. These findings are consistent with those of Jiang et al., who showed that protein information could explain genetic disease phenotypes that could not be explained by transcript information alone (39). Additionally, reduced expression of disease-causing proteins can be identified through comparison with healthy controls, and the discovery of downregulated proteins does not necessarily require a cohort. These findings suggest that they can be applied in the clinical setting for diagnosing a single patient.

Another important finding was that target enrichment of RNA-seq allowed us to identify the genomic basis of an ADA2-deficient case. The expression levels of aberrant transcript was very low due to mRNA instability; Leafcutter results show that the number of aberrant splicing reads is only 0.008% of the cluster. However, target enrichment increased the read depth and revealed the aberrant splicing with intronic variant. These results reflect those of Gildea et al. who also found that target RNA-seq method increased the efficiency of identification of rare splice isoforms, which was difficult with standard RNA-seq (40). Given that the guidelines from ACMG state that a null variant in a gene where loss of function (LOF) is a known mechanism of pathogenicity is the strongest evidence of pathogenesis (41), integrated analysis of T-RNA-seq and proteomics provides significant support for genetic diagnosis by detecting an aberrant splicing and reduced protein levels. In addition, integrated analysis can be a useful tool for the diagnosis of IEI because more than 75% of the known IEI variants show autosomal recessive or X-linked recessive inheritance and are considered LOF (2). Taken together, this study contributes to the clinical management of IEI by providing a rationale for essential specific treatment options, such as TNF inhibitors for ADA2 deficiency (42), abatacept for LRBA deficiency (43), and HSCT for XIAP deficiency.

As mentioned in the literature review, lymphocyte subset analysis, which provides the initial evidence of immune system insufficiency, is a fundamental diagnostic approach for IEI, along with genetic testing (44). We classified all cases into two groups based on the profiles of three proteins specific to B- and T cells and performed DEA to explore immune cell defects. The results of GO analysis for B cells are reasonable, with many proteins involved in B-cell function showing decreased expression. Interestingly, patient B1_P17, clinically diagnosed with late-onset combined immunodeficiency, was assigned to the B-cell-deficient cluster, even though the CD19(+)-B-cell abundance in the peripheral blood was 13.1% and no reduction was observed by FCM. These results further support the suitability of proteomics for IEI diagnosis, as its unbiased comprehensiveness provides a quantitative and functional information regarding immune cell status. However, the B-cell-deficient cluster of T-RNA-seq showed no decreased expression in *AICDA*. This rather contradictory result may be due to inadequate target enrichment of *AICDA*; in fact, some cases showed missing values. In contrast to B-cell analysis, T-cell analysis showed that the proteins involved in ribosome biogenesis and ribosomal RNA processing were downregulated to the same extent as those involved in T-cell function. However, paradoxically, these results coincide with those of well-regarded studies indicating that T-cell activation via T-cell receptor signaling enhances ribosome biosynthesis (45, 46); in other words, T-cell dysfunction inhibits ribosome biogenesis. Overall, these findings suggest that comprehensive proteomics provides insight into not only quantitative abnormalities of immune cells but also the functional aspects of immune cells based on quantitative changes in the molecules involved in their cellular function.

Even though the data processing yielded optimized proteome data, the presence of nonnegligible numbers of MVs remains the major limitation of this study. Seven ineligible cases, which were PCA outliers, were excluded to ensure protein coverage of the data, but 2143 proteins (27% of the total) were excluded due to the large number of samples containing MVs for that protein. Moreover, these proteins included 85 genes covered in the T-RNA-seq (decreasing the total from 399 to 314 genes), which may have caused some bias in the results of correlation analysis. Additionally, analyzing only at a one-time point may underestimate the correlation as proteins and mRNAs have different temporal contexts (47, 48). In part, this is why it is important to analyze protein and mRNA in an integrated manner. Another potential weakness of this study is that proteomic analysis cannot be directly linked to genetic diagnosis when disease-causing proteins show no quantitative changes. In such cases, the changes in the molecules associated with the pathogenic protein could provide the initial clues to the pathogenesis of the disease. However, we did not find such results in the current study. Despite these limitations, this study indicates that integrated analysis of PBMCs is a novel and valuable diagnostic tool for IEI to identify immune cell dysfunction that reflects disease pathogenesis and, in several cases, disease-causing proteins. Further improvements in proteomics data analysis and measurement sensitivity, in combination with its use in multilayered expression analysis with RNA-seq, will contribute to increases in diagnostic yield and a deeper understanding of IEI.

## Materials and methods

### Clinical samples

Seventy IEI patients were recruited from five institutions in three cohorts, with 34, 28, and 8 patients, respectively. In addition, six HCs participated in another period. Throughout this paper, we refer to the cohorts as Batch1 (B1), Batch2 (B2), Batch3 (B3), or Ctrl (C), and patients are identified by group and a unique ID, for example, B1_P1, B2_P35, or B3_P63. Clinical information, such as classification from IUIS, presumptive diagnosis, and candidate genes, was obtained from clinicians. The primary inclusion criterion for IEI patients was the lack of genetic diagnosis via a canonical diagnostic approach such as WES or T-NGS; that is, patients without pathogenic variants in genes consistent with their clinical features and mode of inheritance, and the interpretation of “pathogenic” was according to the ACMG criteria (41). Therefore, when we identified no pathogenic variants, we designated them as “no candidate.” On the other hand, when we identified variants that matched the clinical characteristics but did not meet the ACMG criteria or the mode of inheritance, we designated the gene as a “candidate gene.”

The local ethics boards approved this study of Hiroshima University, Tokyo Medical and Dental University, National Defense Medical College, Gifu University, and Kyoto University.

### Sample preparation

Methods for sample preparation are described in “SI methods.”

### Proteomics and targeted RNA sequencing

Methods for Mass spectrometry-based proteomics and T-RNA-seq are described in “SI methods.”

### Integrated proteomics and targeted RNA sequencing analysis

To understand the etiology and pathogenesis of IEI, we carried out three different approaches using R v4.1 and Bioconductor v3.14 packages.

### Comparison of proteomics and targeted RNA sequencing in genetic diagnosis for inborn errors of immunity

First, to assess whether proteomic data could contribute to the genetic diagnosis, we examined changes in the abundance of proteins encoded by candidate genes in individual cases and compared these results with those of T-RNA-seq. It was impossible to investigate the DEA by comparing individual cases and HC because statistical significance is not a logical criterion in a single-case situation. Therefore, we analyzed the distribution of the protein abundance and the quantitative differences were calculated using *z*-scores. The absolute value of the *z*-score greater than two was defined as significant change. The absolute value of the *z*-score greater than or equal to 2 was defined as significant change. We also analyzed the quantitative differences between each case and the HCs to obtain further information about the biological significance. We calculated the log fold-change (LFC) and mean expression values using limma (49) and visualized the data using ggplot2 (R package). We also used Integrative Genomics Viewer (IGV) v2.8.7 (50) to visualize aligned reads to detect sequence variants and allele-specific expression in T-RNA-seq.

### Correlation analysis of proteomics and targeted RNA sequencing

Second, we examined the discrepancy between protein and mRNA expression levels. Based on the gene profiles identified by both proteomics and T-RNA-seq in 63 of the cases analyzed, the protein–mRNA correlation for each gene was analyzed using Spearman’s correlation coefficient. In addition, the correlation coefficients of genes specific to B, T, and NK cells were compared for later exploratory analysis. Cell-specific proteins were obtained from the database of Immune Cells (51) in The Human Protein Atlas (52). The degree of correlation was set as follows based on the absolute value of the correlation coefficient: 0.7 or higher is strong, 0.4 to 0.7 is moderate, 0.2 to 0.4 is weak, and 0.2 or lower is no correlation.

### Exploratory analysis of B- and T-cell deficiency

Finally, we conducted an exploratory process to identify B-cell- or T-cell-deficient populations. In proteomic analysis, three cell-specific proteins were selected according to the following criteria: (i) proteins with higher specificity and (ii) proteins without MVs or with fewer MVs. In T-RNA-seq, on the other hand, the analysis was based on gene profiles selected based on the criteria described in (i), since T-RNA-seq data are already target-enriched and contain no MVs. We then normalized the data with the *z* score using Genefilter (53), and performed a heatmap analysis of *k*-means clustering using ComplexHeatmaps (54). The *k* value was set to two to discriminate the data points into cell deficiency clusters and others, and the results of proteomics and T-RNA-seq were compared. The validity of the *k*-value was examined by PCA and the elbow method, which determines the optimal number of clusters. We performed differential expression analysis (DEA) on the comprehensive proteomic data to further validate the clustering results. DEA was compared in the cell-deficient cluster vs. others and was performed using DEP (55), which borrows its statistical models from limma (49). In the DEP results, *P* values of <0.05 and LFC of <−1.5 were set as the thresholds for significant differential expression. We then performed Gene Ontology (GO) enrichment analysis of significantly suppressed proteins using ClusterProfiler (56). GO terms related to biological processes were selected, and those with adjusted *P* values below 0.01 were considered significant.

## Supplementary Material

pgad104_Supplementary_Data

## Data Availability

The proteomic data underlying this article are available in [ProteomeXchange] at [http://www.proteomexchange.org], and can be accessed with [PXD038352]. And other data and codes used in this study are described in *SI Appendix*.
