# Correction to: A complementary approach for genetic diagnosis of inborn errors of immunity using proteogenomic analysis

**DOI:** 10.1093/pnasnexus/pgad373

**Published:** 2023-11-15

This is a correction to: Fumiaki Sakura, Kosuke Noma, Takaki Asano, Kay Tanita, Etsushi Toyofuku, Kentaro Kato, Miyuki Tsumura, Hiroshi Nihira, Kazushi Izawa, Kanako Mitsui-Sekinaka, Ryo Konno, Yusuke Kawashima, Yoko Mizoguchi, Shuhei Karakawa, Seiichi Hayakawa, Hiroshi Kawaguchi, Kohsuke Imai, Shigeaki Nonoyama, Takahiro Yasumi, Hidenori Ohnishi, Hirokazu Kanegane, Osamu Ohara, Satoshi Okada, A complementary approach for genetic diagnosis of inborn errors of immunity using proteogenomic analysis, *PNAS Nexus*, Volume 2, Issue 4, April 2023, pgad104, https://doi.org/10.1093/pnasnexus/pgad104

During a retroactive audit conducted by *PNAS Nexus*, it was discovered that this paper was missing a statement acknowledging compliance with the *PNAS Nexus* Human and Animal Participants and Clinical Trials policy:

The local ethics boards at Hiroshima University, Tokyo Medical and Dental University, National Defense Medical College, Gifu University, Kyoto University, and Kazusa DNA Research Institute approved this study. Informed consent was obtained from all participants.

This error has been corrected in the original article.

