# Supplementary material for: A complementary approach for genetic diagnosis of inborn errors of immunity using proteogenomic analysis
Source: PNAS Nexus. 2023 Mar 28;2(4):pgad104. doi: 10.1093/pnasnexus/pgad104 (PMC10109033; doi:10.1093/pnasnexus/pgad104)
Supplement: pgad104_Supplementary_Data [file pgad104_supplementary_data.zip › Supplementary_manuscript_edit.pdf]

Supporting Information for

A complementary approach for genetic diagnosis of inborn errors of immunity using proteogenomic analysis

Fumiaki Sakura, Kosuke Noma, Takaki Asano, Kay Tanita, Etsushi Toyofuku, Kentaro Kato, Miyuki Tsumura, Hiroshi Nihira, Kazushi Izawa, Kanako Mitsui-Sekinaka, Ryo Konno, Yusuke Kawashima, Yoko Mizoguchi, Shuhei Karakawa, Seiichi Hayakawa, Hiroshi Kawaguchi, Kohsuke Imai, Shigeaki Nonoyama, Takahiro Yasumi, Hidenori Ohnishi, Hirokazu Kanegane, Osamu Ohara, and Satoshi Okada

Satoshi Okada
E-mail: sokada@hiroshima-u.ac.jp
Osamu Ohara
E-mail: ohara@kazusa.or.jp

This PDF file includes:

- Supporting text
- Figs. S1 to S6
- Tables S1 to S5
- Legends for Dataset S1 to S6
- SI References

Other supporting materials for this manuscript include the following:

- Datasets S1 to S6

Supporting Information Text

Methods

Sample preparation. PBMCs were isolated from EDTA-coated fresh peripheral blood using density-gradient centrifugation, and red blood cells (RBCs) were lysed with an erythrocyte lysis reagent. The cells were washed in PBS and centrifuged at 300 $\times$g three times. Then the cells were resuspended with TRIzol reagent (Invitrogen, Carlsbad, CA, USA) and stored at -80 $^{\circ}$C for further analysis.

Mass spectrometry-based proteomic. Proteins and RNAs were isolated from samples containing TRIzol according to a previously described method(1). The protein fraction was washed twice with 0.8 mL of acetonitrile and then dissolved in 0.5% sodium dodecanoate and 100 mM Tris-HCl, pH 8.5, using a water bath-type sonicator (Bioruptor II, Cosmo Bio, Tokyo, Japan). Pretreatment for shotgun proteomic analysis was performed as previously reported(2). For LC separation, mobile phases consisted of 0.1% (v/v) formic acid as solvent A and 0.1% (v/v) formic acid/80% (v/v) acetonitrile as solvent B. Each peptide sample (200 ng) was directly injected onto a 75 μm $\times$ 12 cm-nanoLC nano-capillary column (Nikkyo Technos Co., Ltd., Tokyo, Japan) at 40 $^{\circ}$C and then separated with an 80 min gradient at a flow rate of 200 nl/min using an UltiMate 3000 RSLCnano LC system (Thermo Fisher Scientific). Peptides eluting from the column were analyzed on a Q Exactive HFX (Thermo Fisher Scientific) for overlapping window DIA-MS(2, 3). MS1 spectra were collected in the range of 495-785 m/z at 30,000 resolution to set an automatic gain control (AGC) target of 3×10^6 and maximum injection time of 55. MS2 spectra were collected in the range of more than 200 m/z at 30,000 resolution to set an AGC target of 3×10^6, maximum injection time of auto, and stepped normalized collision energy of 22%, 26%, and 30%. The isolation width for MS2 was set to 4 m/z, and overlapping window patterns in 500-780 m/z were used window placements optimized by Skyline v4.1(4). MS files were searched against a human spectral library using Scaffold DIA (Proteome Software, Inc., Portland, OR) as previously reported(5).

Initial processing of the proteomic data. To assess the eligibility of the population, we carried out principal component analysis (PCA) using PCAtools(6). Highly abundant and specific proteins of RBCs and plasma (Dataset S1), which were exemplified in a report by Byrk(7) and Lan(8), were excluded from the dataset because they impaired the analysis of the abundance of residual proteins of interest. Prior to handling missing values (MVs), we assessed the relationship between the protein abundance and MVs with the Pearson correlation coefficient to interpret the nature of the MVs. We then excluded proteins that showed MVs in more than 50% of cases to validate the eligibility of the data, and the remaining MVs were replaced with numerical values using NAguideR(9). To suppress batch effects, we performed normalization using NormalyzerDE(10), which considers several normalization methods and suggests the most effective method for controlling sample dispersion.

Procedures for targeted RNA sequencing. RNA was extracted and recovered from TRIzol using conventional methods. After the quantification and quality control of RNA on an Agilent 2100 Bioanalyzer (Agilent Technologies, Santa Clara, CA, USA), an adjusted amount of total RNA with RNA integrity numbers of 3 to 10 was treated with the NEBNext rRNA Depletion Kit (New England Biolabs, Ipswich, MA, USA) to deplete ribosomal RNA. Then, the NEBNext Ultra II Directional RNA Library Prep Kit for Illumina (New England Biolabs, Ipswich, MA, USA) was used for library preparation. In this procedure, cDNA libraries were captured and amplified for nine cycles with 527 targeted genes, including known IEI genes and PBMC markers (Dataset S6). After recovering the enriched T-RNA-seq libraries, the samples were run on a HiSeq2500 (Illumina, San Diego, CA, USA) in 50-nucleotide single-end mode. A mean read depth of approximately 20 million reads per sample was generated and stored in FASTQ format.

Preprocessing for targeted RNA sequencing data analysis. The FASTQ files were quality controlled and trimmed by sickle(11), then aligned to the GENCODE human reference genome GRCh38.p13 with STAR aligner v2.6(12). Reads were quantified with RSEM v1.3.3(13) to obtain the feature read counts per gene, and the identified genes were refined to the targeted 527 genes. After filtering the targeted genes based on the total read counts, we normalized the data for downstream analysis (Dataset S1).

Detection of splicing outliers in targeted RNA sequencing. Splicing outliers were disclosed using LeafCutter(14) with four inconclusive cases as controls and then visualized with LeafViz(14). LeafCutter utilizes a Dirichlet-multinomial generalized linear model to identify differential splicing based on read counts in an intron cluster. The percentage spliced index (PSI) was used to measure the relative expression of transcript isoforms within intron clusters. The change in PSI (ΔPSI) was used to quantify the relative differential expression of each transcript isoform between the case and controls.

Flow cytometry analysis of CTLA4. PBMCs were isolated from the patient with LRBA deficiency and the healthy control and resuspended at 1×10^6 /ml in RPMI medium with 10% fetal bovine serum. Cells were then incubated with anti-CD3/CD28 antibodies (Miltenyi Biotec, Bergisch Gladbach, Germany) for 16 hrs. After incubation, cells were washed and stained with anti-CD4-FITC (BioLegend, San Diego, CA, USA). Following fixation and permeabilization, cells were stained with anti-FOXP3-Alexa Fluor 647 (BioLegend) and anti-CTLA4-PerCP (BioLegend). Data were collected with a BD FACSVerse (BD Biosciences, Franklin Lakes, NJ, USA) and analyzed with FlowJo software (BD Life Sciences).

Western blotting of LRBA. Proteins lysates from PBMCs of the patient and healthy control were separated on 10% acrylamide gel, followed by transfer onto the PVDF membrane. LRBA was detected using the primary antibody of polyclonal rabbit anti-LRBA/BGL (Abcam, Kenbridge, UK) and the secondary antibody of ECL anti-rabbit IgG (GE Healthcare, Uppsala, Sweden). Beta-actin was used as a loading control.

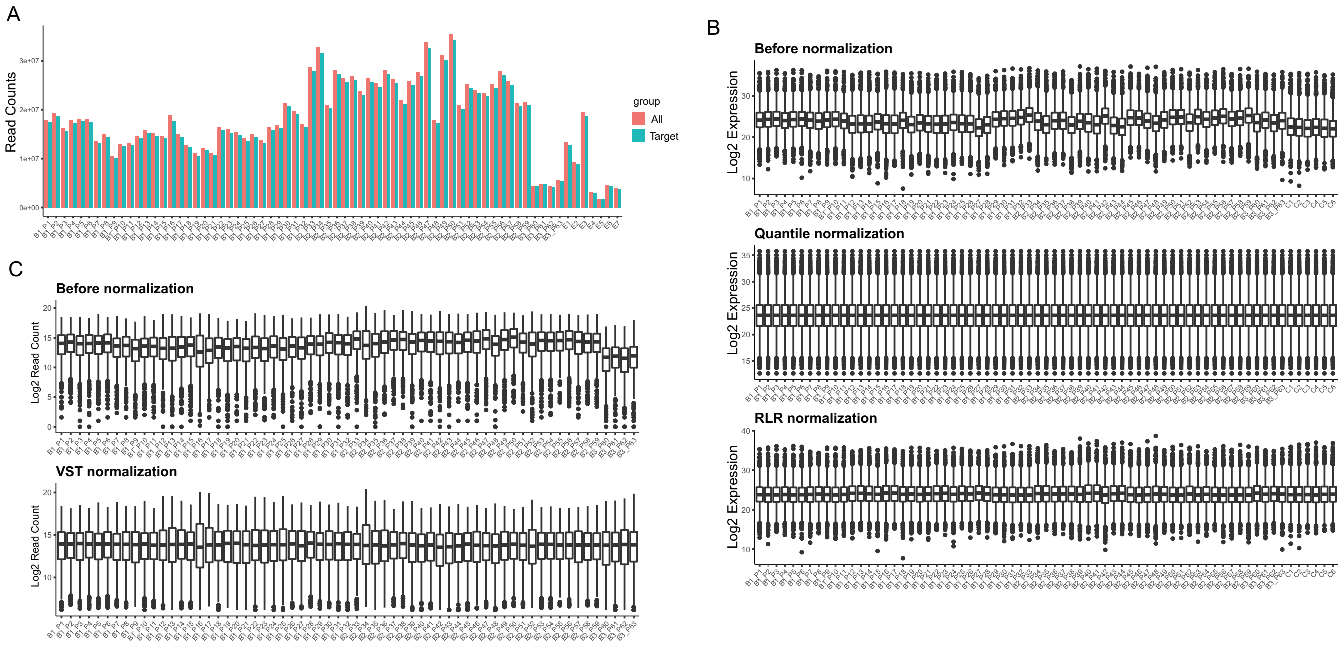

Fig. S1. Interpretation and optimization of the data. (A) The efficiency of target enrichment in RNA-seq. Red bars show total read counts, and blue bars show targeted read counts. (B) Comparison of the distribution of expression profiles in raw and normalized proteomic data (top; Raw data, middle; Quantile normalization, bottom; Robust Linear Regression normalization). (C) Comparison of the distribution of expression profiles in raw and normalized T-RNA-seq data (top; Raw data, bottom; variance stabilizing normalization).

NM_001287345.2: c.-196+1G>T

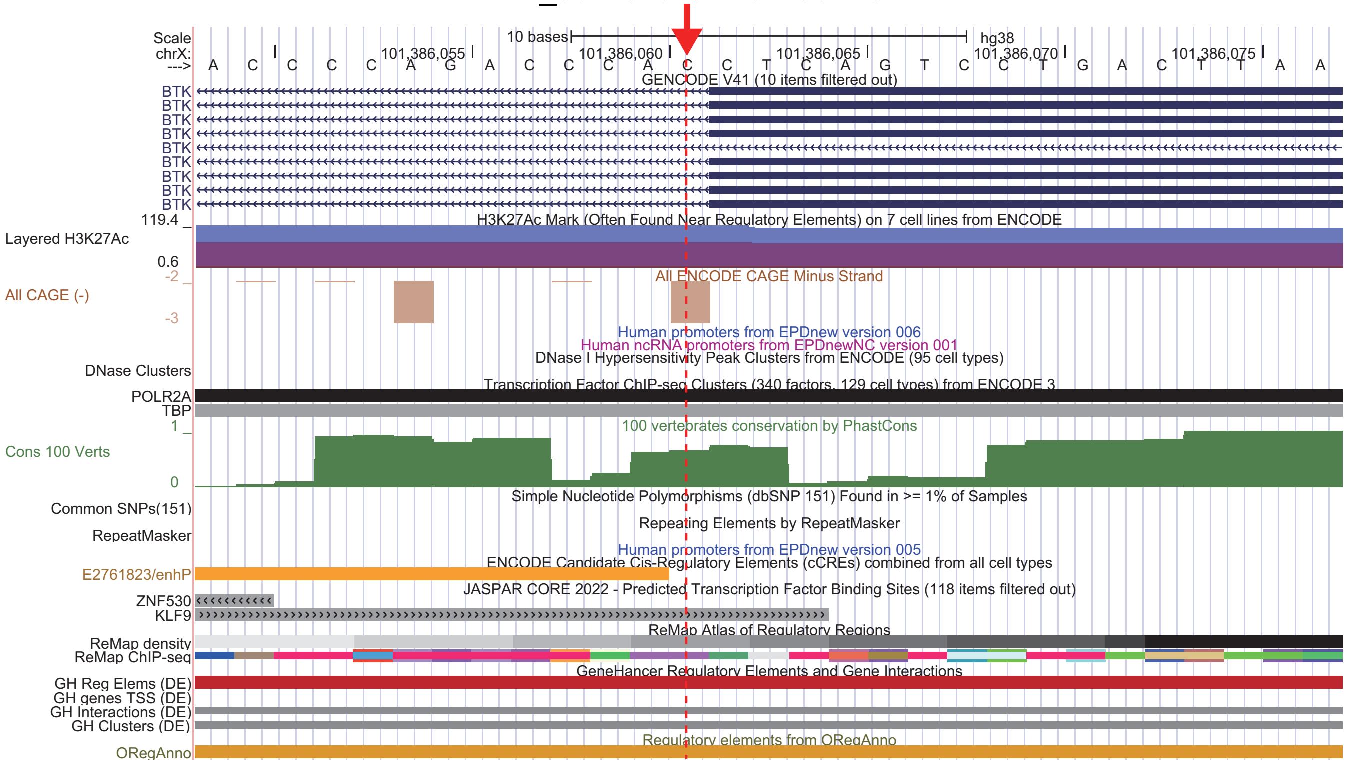

Fig. S2. UCSC Genome Browser view for the splice-site variant in *BTK* (GRCh38/hg38). The red arrow and dashed line indicate the position of c.-196+1. The GENCODE Genes track of *BTK* is shown in the top panel. Below that, transcriptional regulatory elements from the database of ENCODE, EPDnew, GeneHancer, JASPARCORE 2022, ORegAnno, and ReMap Atras are shown. Green bars in the center track indicate evolutionary conservation in 100 vertebrate species.

A

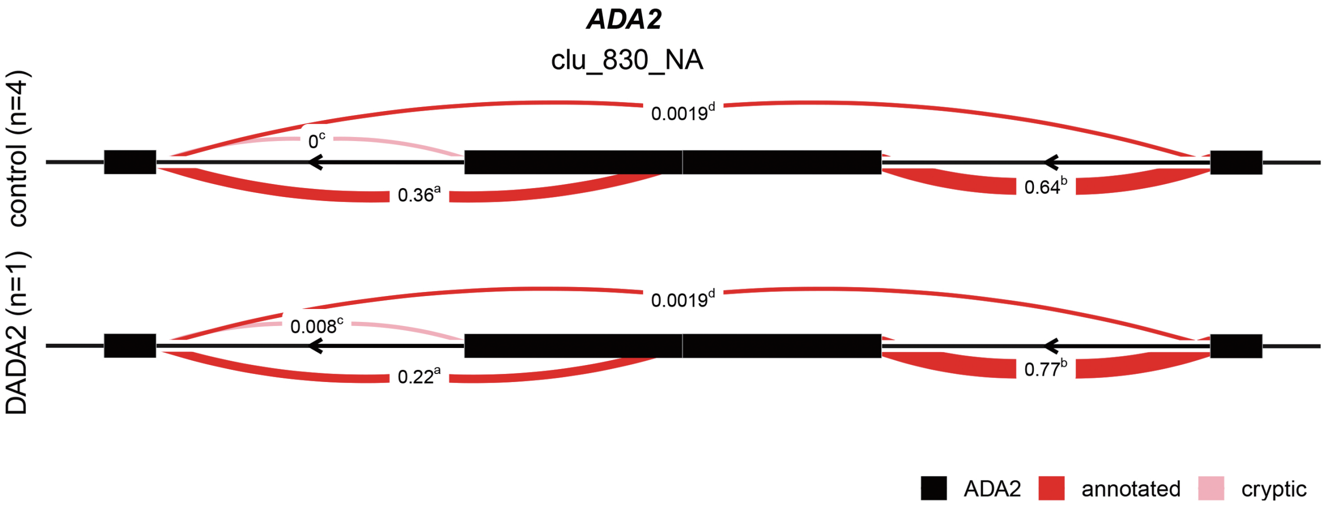

	chr	start	end	verdict	dPSI
a	chr22	17188447	17189942	annotated	−0.133
b	chr22	17190032	17191683	annotated	0.125
c	chr22	17188447	17189840	cryptic_fiveprime	0.008
d	chr22	17188447	17191683	annotated	−0.001

B

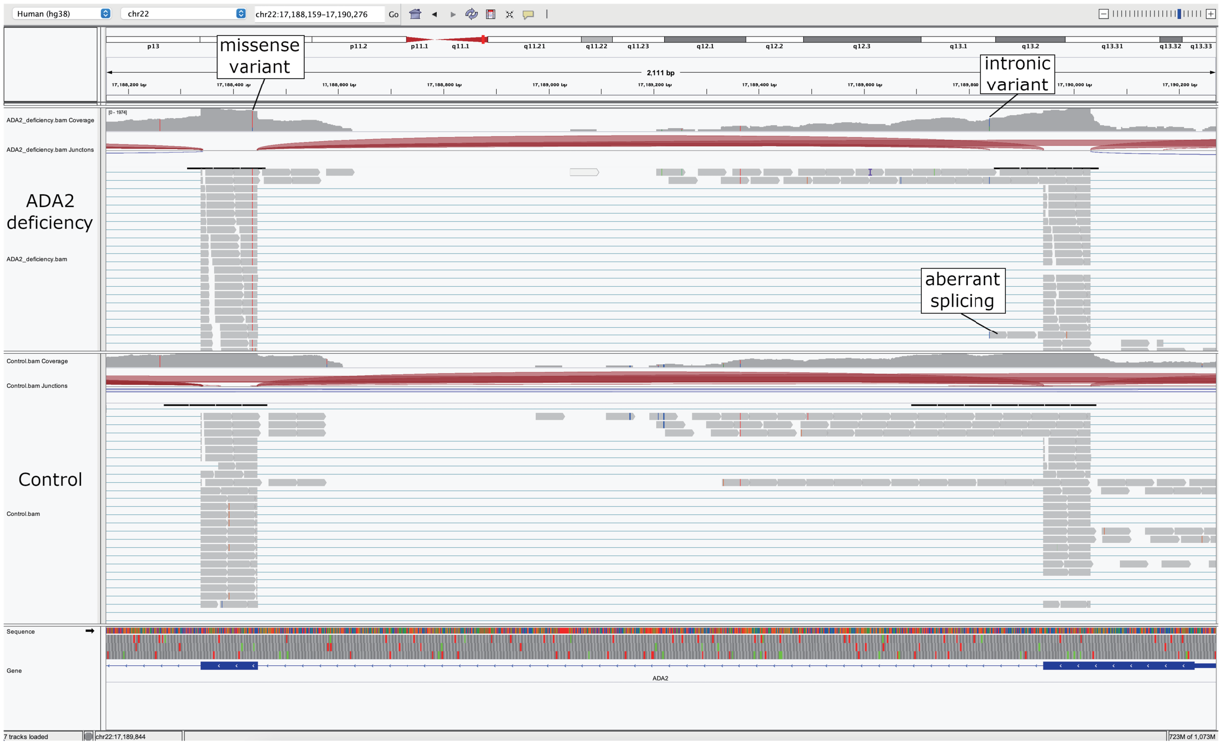

Fig. S3. Abnormal findings of T-RNA-seq in the patient wiht ADA2 deficiency. (A) Visualization of differential splicing in comparison with ADA2 deficiency and four controls. The red lines indicate splicing junctions connecting to the exons (shown in black). All junctions are annotated with percentage spliced index (PSI), reflecting the ratio of junction reads within the cluster. The PSI value of controls is 0 in the cryptic junction (shown in pink), which means that the junction is specific to the patient with ADA2 deficiency. (B) Visualization of the abnormal findings in T-RNA-seq by IGV. Heterozygous missense variant shows allele-specific expression in T-RNA-seq, with the variant allele accounting for 80% of all reads and the wild type for 20%. Abnormal splicing harbors a heterozygous intron variant at the 5' end, generating an inappropriate donor site. Intronic and missense variants are carried by different reads, indicating compound heterozygosity.

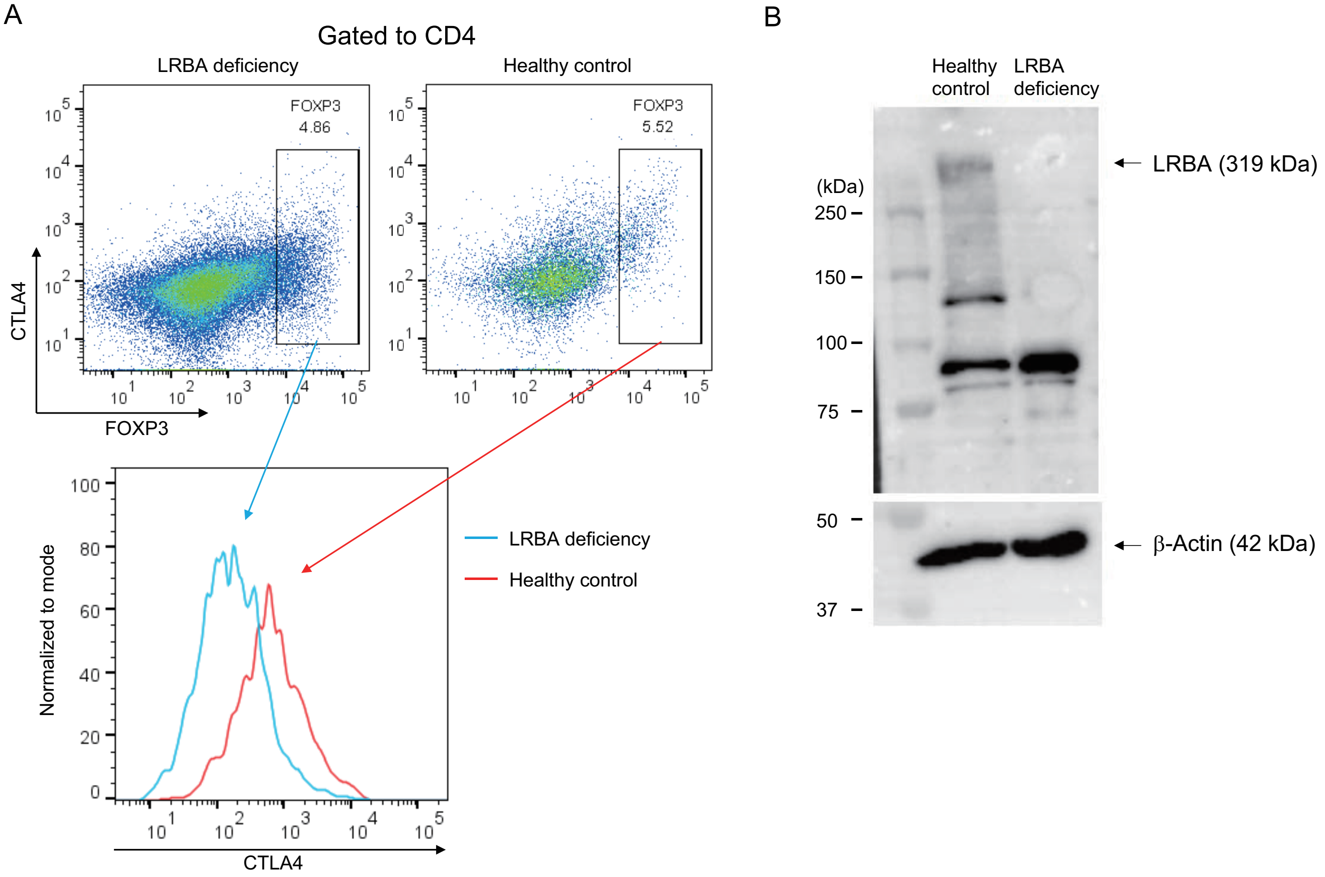

Fig. S4. Laboratory findings of an LRBA-deficient case. (A) CTLA4 expression on CD4+FOXP3+ Tregs in flow cytometric analysis. The upper panel shows the expression of CTLA4 and FOXP3 in PBMCs stimulated with anti-CD3/CD28 antibodies. The bottom panel shows the reduced CTLA4 expression of Tregs in the patient compared to the healthy control. (B) LRBA expression in Western blotting. LRBA protein (319 kDa) is not detected on PBMCs in the patient compared to the healthy control.

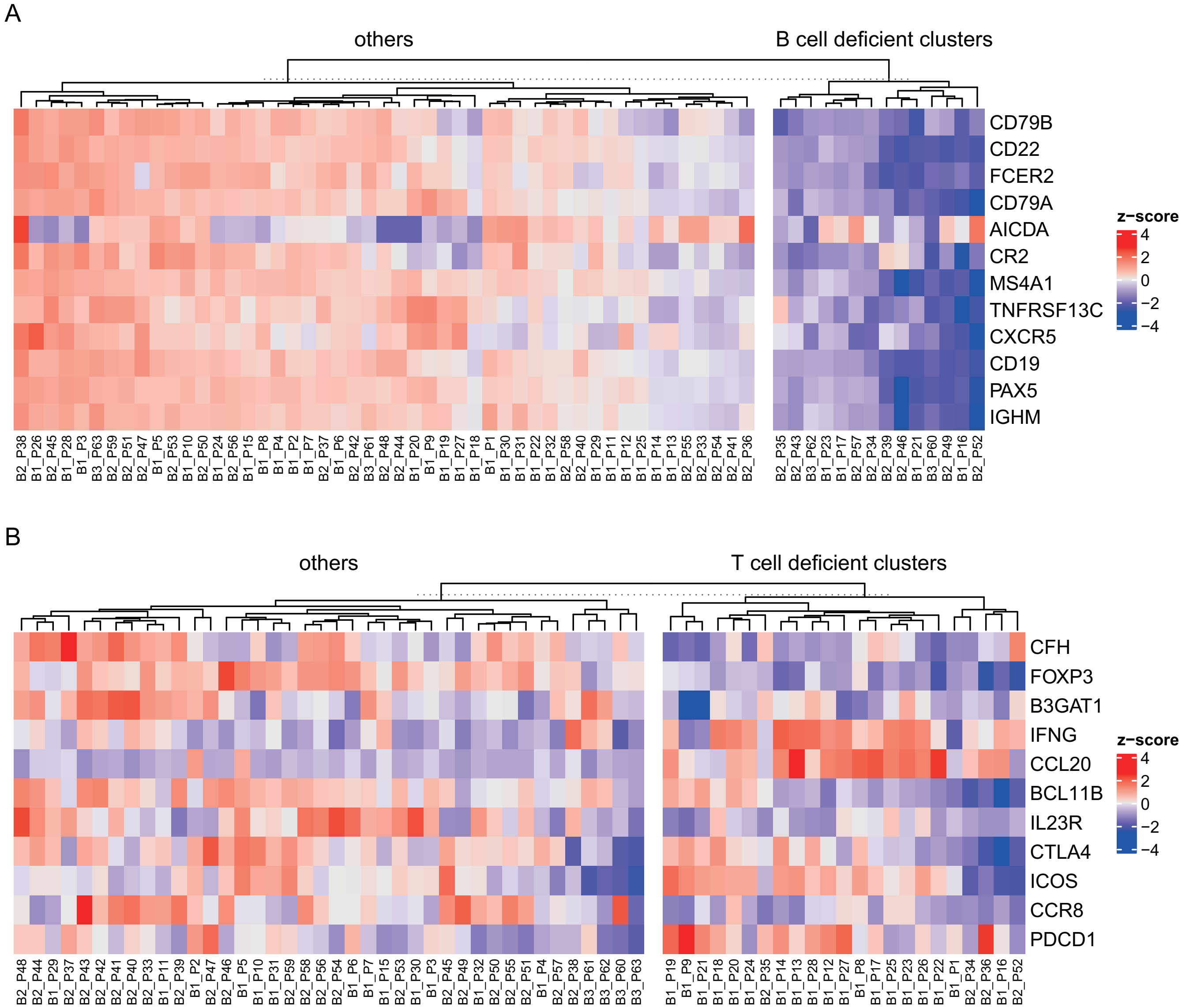

Fig. S5. Hierarchical clustering of B- and T-cell deficiency in T-RNA-seq. (A) The heatmap of k-means clustering shows cluster segregation with decreased expression of B-cell-specific genes. The color scale reflects the z score, with red indicating a positive value and blue a negative value. (B) The heatmap of k-means clustering of T-cell-sepecific genes in T-RNA-seq.

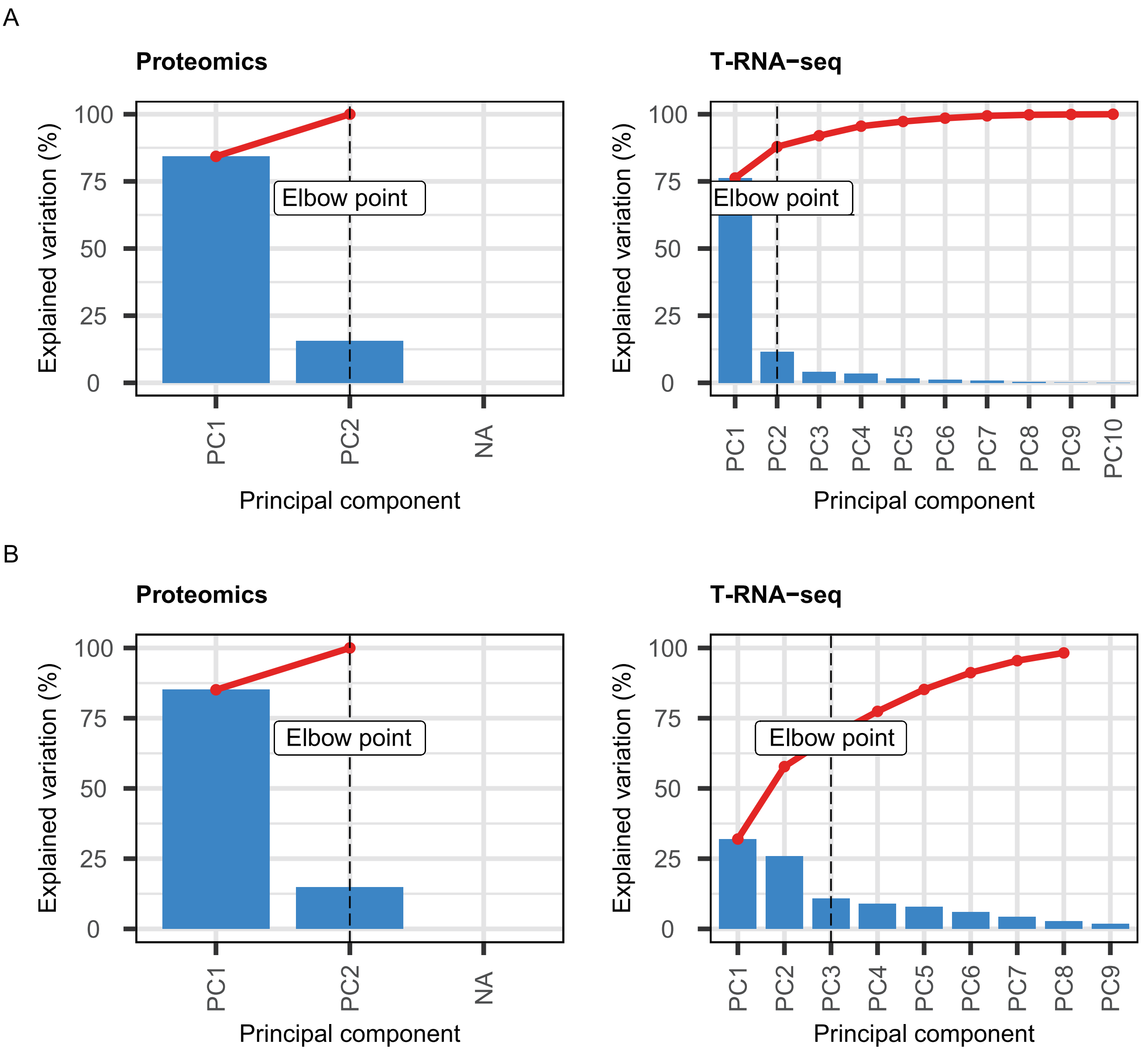

Fig. S6. Comparison of clustering analysis of B-cell and T-cell in proteomics and T-RNA-seq. (A) Scree plot of principal components as criteria for clustering in B-cell. Throughout all figures, the x-axis shows all principal components, and the y-axis shows the proportion of the variable explained by each principal component. The broken red line indicates the cumulative proportion of the variables explained by the principal components. The elbow point suggests the optimal number of clusters, which means the optimal k-value in the k-means method. (B) Scree plot of principal components as criteria for clustering in T-cell. The elbow point in RNA-seq is the fourth principal component (all other elbow points are second principal components)

Table S1. Characteristics of patients and the result of prior genetic studies

Patient ID	IUIS classification	Clinical diagnosis	Clinical phenotypes	Genomic analysis	Candidate genes (inheritance mode)	Variants (ACMG Classification)
B1_P1	4	Immune dysregulation	Thrombocytopenia, Splenomegaly	WES	No candidate	N.A.
B1_P2	3	CVID	Hypogammaglobulinemia, Atopic dermatitis, asthma	WES	No candidate	N.A.
B1_P3	3	CVID	Hypogammaglobulinemia	WES	No candidate	N.A.
B1_P4	3	CVID	Hypogammaglobulinemia, ADEM, Recurrent infection	WES	No candidate	N.A.
B1_P5	7	Autoinflammation	Recurrent fever, Abdominal pain	WES	No candidate	N.A.
B1_P6	7	Autoinflammation	Recurrent fever, Hepatitis	WES	No candidate	N.A.
B1_P7	4	Lymphomatoid granulomatosis	Chronic EBV infection, Polyneuropathy	WES	No candidate	N.A.
B1_P8	1	LOCID	Hypogammaglobulinemia	WES	No candidate	N.A.
B1_P9	4	Immune dysregulation	B-LPD	WES	No candidate	N.A.
B1_P10	3	CID	Recurrent infection, T-cell dysfunction	WES	No candidate	N.A.
B1_P11	3	CVID	Hypogammaglobulinemia	WES	No candidate	N.A.
B1_P12	3	CVID	Hypogammaglobulinemia	T-NGS	No candidate	N.A.
B1_P13	3	CVID	Hypogammaglobulinemia	WES	No candidate	N.A.
B1_P14	1	LOCID	Hypogammaglobulinemia	T-NGS	No candidate	N.A.
B1_P15	3	CVID	Hypogammaglobulinemiaa	WES	No candidate	N.A.
B1_P16	3	CVID	Idiopathic enteritis	T-NGS	No candidate	N.A.
B1_P17	1	LOCID	Hypogammaglobulinemia	T-NGS	No candidate	N.A.
B1_P18	3	CVID	Hypogammaglobulinemia	WES	No candidate	N.A.
B1_P19	3	CVID	Hypogammaglobulinemia	WES	No candidate	N.A.
B1_P20	3	CVID	Hypogammaglobulinemia	WES	No candidate	N.A.
B1_P21	3	XLA (BTK deficiency)	Hypogammaglobulinemia	WES	No candidate	N.A.
B1_P22	4	XIAP deficiency	Recurrent HLH	T-NGS	No candidate	N.A.
B1_P23	3	CVID	Hypogammaglobulinemia	T-NGS	No candidate	N.A.
B1_P24	3	CVID	Hypogammaglobulinemia	WES	No candidate	N.A.
B1_P25	3	CVID	Hypogammaglobulinemia	WES	No candidate	N.A.
B1_P26	2	EDA-ID	Ectodermal dysplasia	WES	No candidate	N.A.
B1_P27	3	CVID	Hypogammaglobulinemia	WES	No candidate	N.A.
B1_P28	1	CID	EBV-lymphoma	WES	No candidate	N.A.
B1_P29	7	Autoinflammation	Cerebral infraction	T-NGS	*ADA2* (AR)	c.982G>A hetero (VUS, but known pathogenic(15))
B1_P30	7	Juvenile Behçet's disease	Aphthous stomatitis	WES	No candidate	N.A.
B1_P31	4	Immune dysregulation	No data	WES	No candidate	N.A.
B1_P32	7	Autoinflammation	Recurrent fever, Aseptic meningitis	WES	No candidate	N.A.
B2_P33	3	CVID	Hypogammaglobulinemiaa, Recurrent infection	T-NGS	No candidate	N.A.
B2_P34	3	APDS	Hypogammaglobulinemia	T-NGS	No candidate	N.A.
B2_P35	4	LRBA deficiency	AIHA, AIN, ITP	WES	*LRBA* (AR)	c.1219_1220delTT hetero (Likely Pathogenic)
B2_P36	4	IBD	Abdominal pain, Recurrent diarrhea	T-NGS	No candidate	N.A.
B2_P37	1	SCID	Recurrent infection	WES	No candidate	N.A.
B2_P38	6	MSMD	BCG myelitis	WES	No candidate	N.A.
B2_P39	3	CVID	Hyper IgM, ITP, SLE	WES	*TCF3* (AD, AR)	c.319G>A hetero (VUS) / c.1592C>T hetero (Benign)
B2_P40	3	CVID	Hypogammaglobulinemia	T-NGS	No candidate	N.A.
B2_P41	3	CVID	Hypogammaglobulinemia	T-NGS	No candidate	N.A.
B2_P42	3	CVID	Hypogammaglobulinemia	T-NGS	No candidate	N.A.
B2_P43	3	CVID	Hypogammaglobulinemia	T-NGS	*TRAF3* (AD)	c.1415C>T hetero (VUS)
B2_P44	3	CVID	Hypogammaglobulinemia	T-NGS	*MSH2* (AD)	c.118G>A hetero (VUS)
B2_P45	2	MOPD1	Hypogammaglobulinemia	T-NGS	No candidate	N.A.
B2_P46	3	CVID	Hypogammaglobulinemia	T-NGS	No candidate	N.A.
B2_P47	3	CVID	Hypogammaglobulinemia	T-NGS	No candidate	N.A.
B2_P48	3	CVID	Hypogammaglobulinemia	T-NGS	No candidate	N.A.
B2_P49	4	CID	Hashimoto thyroiditis, Type 1 diabetes mellitus	WES	No candidate	N.A.
B2_P50	7	CINCA syndrome	Vasculitis, Sensorineural hearing impairment	WES	No candidate	N.A.
B2_P51	7	Interferonopathy	Periodic fever, Asthma	WES	No candidate	N.A.
B2_P52	4	CID	Evans syndrome, B-cell deficiency	WES	No candidate	N.A.
B2_P53	7	Interferonopathy	Periodic fever, Failure to thrive	WES	No candidate	N.A.
B2_P54	7	Autoinflammation	Systemic vasculitis	WES	*PIK3CD* (AD, AR)	c.2689G>A hetero (VUS)
B2_P55	7	Autoinflammation	Unexplained fever	WES	No candidate	N.A.
B2_P56	7	Interferonopathy	Unexplained fever, Spondylitis	WES	No candidate	N.A.
B2_P57	7	JIA	Macrophage activation syndrome	WES	No candidate	N.A.
B2_P58	7	Interferonopathy	Recurrent fever, Aphthous stomatitis	WES	No candidate	N.A.
B2_P59	4	APDS	Hyper IgE syndrome	WES	No candidate	N.A.
B3_P60	3	CVID	Hypogammaglobulinemia, Skeletal dysplasia	WES	No candidate	N.A.
B3_P61	3	CVID	Hypogammaglobulinemia	T-NGS	No cadidate	N.A.
B3_P62	3	CVID	Hypogammaglobulinemia, Abnormal pigmentation	WES	No candidate	N.A.
B3_P63	7	IBD	Multiple intestinal stricture, Interstitial pneumonia	T-NGS	No candidate	N.A.
E1	3	CVID	Hypogammaglobulinemiaa	WES	No candidate	N.A.
E2	3	CVID	Hypogammaglobulinemia	WES	No cadidate	N.A.
E3	3	CVID	Hypogammaglobulinemia, focal epilepsy, ASD	WES	*RTEL1* (AD, AR)	c.3064C>G hetero (VUS)
E4	4	APS	No data	WES	*PEPD* (AR)	c.410C>T hetero (VUS) / c.1291C>T hetero (VUS)
E5	3	LOCID	Reccurent pneumonia	WES	No candidate	N.A.
E6	4	Immune dysregulation	ALPS like	WES	No candidate	N.A.
E7	7	IBD	Recurrent fever	T-NGS	No candidate	N.A.

AR, autosomal recessive; AD, autosomal dominant; VUS, variant of unknown significance

Table S2. IUIS classification, Clinical diagnosis, and Lymphocyte subset in the B-cell-deficient clusters in proteomics

Patient ID	IUIS classification	Clinical diagnosis	B-cell %/lymphocytes	T-cell %/lymphocytes
B1_P16	3	CVID	0	57.2
B1_P17	1	LOCID	13.1	67.4
B1_P21	3	XLA	0.1	65.5
B1_P23	3	CVID	2.2	77.3
B2_P34	3	APDS	2.2	42.5
B2_P39	3	CVID	N.A.	N.A.
B2_P43	3	CVID	0.8	95
B2_P46	3	CVID	N.A.	N.A.
B2_P49	4	CID	N.A.	N.A.
B2_P52	4	CID	N.A.	N.A.
B2_P57	7	JIA	N.A.	N.A.
B3_P60	3	CVID	N.A.	N.A.

Table S3. Characteristics of the B-cell-deficient clusters in T-RNA-seq

Patient ID	IUIS classification	Clinical diagnosis	B-cell %/lymphocytes	T-cell %/lymphocytes
B1_P16	3	CVID	0	57.2
B1_P17	1	LOCID	13.1	67.4
B1_P21	3	XLA (BTK deficiency)	0.1	65.5
B1_P23	3	CVID	2.2	77.3
B2_P34	3	APDS	2.2	42.5
B2_P35	4	LRBA deficiency	7.7	86.1
B2_P39	3	CVID	N.A.	N.A.
B2_P43	3	CVID	0.8	95
B2_P46	3	CVID	N.A.	N.A.
B2_P49	4	CID	N.A.	N.A.
B2_P52	4	CID	N.A.	N.A.
B2_P57	7	JIA	N.A.	N.A.
B3_P60	3	CVID	N.A.	N.A.
B3_P62	3	CVID	N.A.	N.A.

Table S4. IUIS classification, clinical diagnosis, and lymphocyte subset in the T-cell-deficient clusters in proteomics

Patient ID	IUIS classification	Clinical diagnosis	T-cell %/lymphocytes	CD4+ T-cell %/CD3+ T-cell	CD8+ T-cell %/CD3+ T-cell	B-cell %/lymphocytes
B1_P1	4	Immune dysregulation	53.9	58.6	38.0	20.9
B1_P8	1	LOCID	90.4	37.6	54.1	1.1
B1_P12	3	CVID	68.9	57.1	36.3	4.1
B1_P13	3	CVID	73.4	38.6	52.7	9.4
B1_P14	1	LOCID	71.7	78.3	11.4	2.4
B1_P16	3	CVID	57.2	34.1	54.2	0.0
B1_P17	1	LOCID	67.4	61.4	29.9	13.1
B1_P22	4	XIAP deficiency	N.A.	N.A.	N.A.	N.A.
B1_P23	3	CVID	77.3	29.3	66.3	2.2
B1_P25	3	CVID	77.4	38.7	54.0	10.6
B1_P26	2	EDA-ID	33.4	70.3	29.7	32.4
B1_P27	3	CVID	71.4	37.4	42.8	2.0
B1_P28	1	CID	56.8	40.9	56.6	35.6
B2_P34	3	APDS	42.5	42.1	45.8	2.2
B2_P35	4	LRBA deficiency	86.1	37.3	57.1	7.7
B2_P36	4	IBD	83.4	47.8	48.6	6.8
B2_P37	1	SCID	46.8	48.8	41.4	6.3
B2_P40	3	CVID	N.A.	N.A.	N.A.	N.A.
B2_P49	4	CID	N.A.	N.A.	N.A.	N.A.
B2_P52	4	CID	N.A.	N.A.	N.A.	N.A.
B3_P60	3	CVID	N.A.	N.A.	N.A.	N.A.
B3_P62	3	CVID	N.A.	N.A.	N.A.	N.A.
B3_P63	7	IBD	N.A.	N.A.	N.A.	N.A.

Table S5. Characteristics of the T-cell-deficient cluster in T-RNA-seq

Patient ID	IUIS classification	Clinical diagnosis	T-cell %/lymphocytes	CD4+ T-cell %/CD3+ T-cell	CD8+ T-cell %/CD3+ T-cell	B-cell %/lymphocytes
B1_P1	4	Immune dysregulation	53.9	58.6	38.0	20.9
B1_P4	3	CVID	91.6	83.8	11.0	2.7
B1_P8	1	LOCID	90.4	37.6	54.1	1.1
B1_P9	4	Immune dysregulation	N.A.	N.A.	N.A.	N.A.
B1_P12	3	CVID	68.9	57.1	36.3	4.1
B1_P13	3	CVID	73.4	38.6	52.7	9.4
B1_P14	1	LOCID	71.7	78.3	11.4	2.4
B1_P16	3	CVID	57.2	34.1	54.2	0.0
B1_P17	1	LOCID	67.4	61.4	29.9	13.1
B1_P18	3	CVID	84.2	68.9	21.1	0.47
B1_P19	3	CVID	79.0	52.0	42.0	13.6
B1_P20	3	CVID	73.4	62.9	31.5	10.5
B1_P21	3	XLA (BTK deficiency)	65.6	51.8	30.2	0.1
B1_P22	4	XIAP deficiency	N.A.	N.A.	N.A.	N.A.
B1_P23	3	CVID	77.3	29.3	66.3	2.2
B1_P24	3	CVID	73.8	69.1	21.2	20
B1_P25	3	CVID	77.4	38.7	54.0	10.6
B1_P26	2	EDA-ID	33.4	70.3	29.7	32.4
B1_P27	3	CVID	71.4	37.4	42.8	2.0
B1_P28	1	CID	56.8	40.9	56.6	35.6
B2_P34	3	APDS	42.5	42.1	45.8	2.2
B2_P35	4	LRBA deficiency	86.1	37.3	57.1	7.7
B2_P36	4	IBD	83.4	47.8	48.6	6.8
B2_P38	6	MSMD	N.A.	N.A.	N.A.	N.A.
B2_P52	4	CID	N.A.	N.A.	N.A.	N.A.
B3_P62	3	CVID	N.A.	N.A.	N.A.	N.A.
B3_P63	7	IBD	N.A.	N.A.	N.A.	N.A.

SI Dataset S1 (proteome_data.xlsx)
Data and codes of proteomic analysis

SI Dataset S2 (RNA-seq_data.xlsx)
Data and codes of targeted RNA sequencing

SI Dataset S3 (diagnostic_analysis.xlsx)
Data and codes of diagnostic analysis

SI Dataset S4 (protein_RNA_correlation.xlsx)
Data for the correlation analysis of protein and RNA expression levels

SI Dataset S5 (exploratory_analysis.xlsx)
Data and codes of exploratory analysis

SI Dataset S6 (RNAtarget_genes.xlsx)
Targeted genes in RNA sequencing

References

1. Y Kawashima, et al., Proteogenomic Analyses of Cellular Lysates Using a Phenol-Guanidinium Thiocyanate Reagent. *J. Proteome Res.* **18**, 301–308 (2019).
2. Y Kawashima, et al., Optimization of Data-Independent Acquisition Mass Spectrometry for Deep and Highly Sensitive Proteomic Analysis. *Int. J. Mol. Sci.* **20**, 5932 (2019).
3. D Amodei, et al., Improving Precursor Selectivity in Data-Independent Acquisition Using Overlapping Windows. *J. Am. Soc. for Mass Spectrom.* **30**, 669–684 (2019).
4. B MacLean, et al., Skyline: An open source document editor for creating and analyzing targeted proteomics experiments. *Bioinforma. (Oxford, England)* **26**, 966–968 (2010).
5. H Sato, et al., In-Depth Serum Proteomics by DIA-MS with In Silico Spectral Libraries Reveals Dynamics during the Active Phase of Systemic Juvenile Idiopathic Arthritis. *ACS omega* **7**, 7012–7023 (2022).
6. K Blighe, PCAtools: Everything Principal Component Analysis (2022).
7. AH Bryk, JR Wiśniewski, Quantitative Analysis of Human Red Blood Cell Proteome. *J. Proteome Res.* **16**, 2752–2761 (2017).
8. J Lan, et al., Systematic Evaluation of the Use of Human Plasma and Serum for Mass-Spectrometry-Based Shotgun Proteomics. *J. Proteome Res.* **17**, 1426–1435 (2018).
9. S Wang, et al., NAguideR: Performing and prioritizing missing value imputations for consistent bottom-up proteomic analyses. *Nucleic Acids Res.* **48**, e83 (2020).
10. J Willforss, A Chawade, F Levander, NormalyzerDE: Online Tool for Improved Normalization of Omics Expression Data and High-Sensitivity Differential Expression Analysis. *J. Proteome Res.* **18**, 732–740 (2019).
11. najoshi, Sickle - A windowed adaptive trimming tool for FASTQ files using quality (2021).
12. A Dobin, et al., STAR: Ultrafast universal RNA-seq aligner. *Bioinforma. (Oxford, England)* **29**, 15–21 (2013).
13. B Li, CN Dewey, RSEM: Accurate transcript quantification from RNA-Seq data with or without a reference genome. *BMC Bioinforma.* **12**, 323 (2011).
14. YI Li, et al., Annotation-free quantification of RNA splicing using LeafCutter. *Nat. Genet.* **50**, 151–158 (2018).
15. N Keer, M Hershfield, T Caskey, S Unizony, Novel compound heterozygous variants in CECR1 gene associated with childhood onset polyarteritis nodosa and deficiency of ADA2. *Rheumatology* **55**, 1145–1147 (2016).
